# Comparison of meat quality, fatty acid composition and aroma volatiles of Chikso and Hanwoo beef

**DOI:** 10.5713/ajas.17.0902

**Published:** 2018-04-25

**Authors:** Dicky Tri Utama, Chang Woo Lee, Yeon Soo Park, Aera Jang, Sung Ki Lee

**Affiliations:** 1 Department of Applied Animal Science, College of Animal Life Sciences, Kangwon National University, Chuncheon 24341, Korea; 2 Gangwon Province Livestock Research Institute, Hoengseong 25266, Korea

**Keywords:** Aroma, Beef, Chikso, Hanwoo, Meat Quality

## Abstract

**Objective:**

Although Hanwoo has been selected as the superior commercial beef cattle breed in Korea, Chikso (Korean brindle cattle) is still recognized as a valuable breed for beef production. The aim of this study was to compare the meat quality, fatty acid composition and aroma volatiles of beef from Chikso and Hanwoo steers maintained under identical feed management, as information regarding these characteristics is still limited.

**Methods:**

A total of 19 carcasses with a quality grade of 1 were selected, and strip loin (*longissimus lumborum*) cuts were collected from 11 Hanwoo carcasses and 8 Chikso carcasses. Meat quality and aroma analyses were performed at day four postmortem.

**Results:**

Though Hanwoo strip loin tended to have higher fat content (15.37%) than Chikso (12.01%), no significant differences were observed. Meat pH, water-holding capacity, cooking loss, shear force value, instrumental surface color (Commission International De L’eclairage L*, a*, b*, chroma, and hue angle) and fatty acid composition were not significantly different. Roasted Chikso beef released more intense aroma than roasted Hanwoo beef based on the total area units of identified volatiles. Among identified volatiles, the amounts of toluene, heptanal, octanal, and nonanal were higher in roasted Chikso beef than in roasted Hanwoo beef. In addition, the aroma pattern of the roasted beef from these breeds was well-discriminated by electronic nose.

**Conclusion:**

No distinct differences were found in terms of meat quality between Hanwoo and Chikso beef in this study. However, the aroma pattern and volatiles of roasted Hanwoo and Chikso beef were different according to instrumental analysis.

## INTRODUCTION

According to the Domestic Animal Diversity Information System of the United Nations Food and Agriculture Organization (FAO), there are four indigenous cattle breeds in Korea that are classified based on their coat colors, including Hanwoo (brown cattle) and Chikso (brindle cattle) [1]. The unique coat color pattern of Chikso has made it easy to identify among other native Korean cattle. However, the Chikso population has been stable at low numbers and herds can be found in some limited areas [2]. Although Hanwoo has been selected as a major commercial breed in Korea for beef production, Chikso is still recognized as a valuable breed for beef cattle breeding programs.

Among Korean consumers, grain-fed Hanwoo beef is renowned as the most favorable and exclusive type of beef due to its marbling quality compared with the other breeds available in the Korean market [3]. Indeed, breed, diet and the amount of intramuscular fat (IMF) influence meat flavor. With respect to the effect of different breeds on flavor, a study revealed that the beef of grass-fed Wagyu had higher concentrations of volatiles than grass-fed Angus beef. Furthermore, the effect of diet (grain vs grass) revealed that the beef of grass-fed cattle released higher concentrations of volatiles than the beef of grain-fed cattle [4]. To the best of our knowledge, there is no comparison study on aroma volatiles between Hanwoo and Chikso raised under identical management and with similar quality grade carcasses. It is thus necessary to investigate the effect of breed on general meat quality traits and aroma volatiles between Chikso and Hanwoo.

The electronic nose is a sensor array-based robotic system that can overcome the subjectivity of human panelists on assessing food aroma for quality control purposes such as food authenticity [5]. In previous studies, this tool has been used to discriminate meat-based seasonings and beef aroma changes during refrigerated storage [6,7]. Aroma volatile compounds of beef are mainly the products of thermal lipid degradation, particularly heat-induced oxidation of fatty acids and the Maillard reaction between amino acids and reducing sugars [8]. The combination of aroma discrimination by electronic nose and head space (HS) volatile identification by solid phase microextraction-gas chromatography-mass spectrometry has been used in some recent studies on beef products [9,10]. Therefore, the aim of this study was to compare the meat quality, fatty acid composition and aroma volatiles of strip loin from Chikso and Hanwoo steers.

## MATERIALS AND METHODS

### Samples

Hanwoo and Chikso steers (24±1 month old) were finished on a grain-based diet with 12% crude protein and 74% total digestible nutrients for 180 days prior to slaughter at Gangwon Provincial Livestock Research Center’s farm. To avoid an effect on aroma volatiles from quality grade or fat content, strip loin cuts (*longissimus lumborum*) were collected from 11 Hanwoo carcasses and 8 Chikso carcasses with the same quality grade 1. Samples were vacuum packaged and chilled at 2°C±2°C. Meat quality and aroma analyses were performed at day 4 postmortem.

### Proximate and fatty acid composition analysis

Samples were ground using a food blender at minimum speed for 10 s (HMF-1600PB, Hanil Electronics Co., Ltd., Seongnam, Korea). Proximate composition was determined by AOAC official methods [11]. Moisture content was determined by drying the samples in the oven at 105°C for 24 h. Crude fat content was determined by ether extraction using the Soxhlet system. Nitrogen content was determined using the Kjeltec system (2200 Kjeltec Auto Distillation Unit, Foss, Hillerød, Denmark), and crude protein was calculated as nitrogen content multiplied by 6.25. Crude ash was determined by burning the samples in the muffle furnace at 550°C for 12 h.

An Agilent gas chromatography system (6890N, Agilent Technologies, Santa Clara, CA, USA) with an auto sampler (7683, Agilent Technologies, USA) was used for determining fatty acid composition. The fat was extracted from ground meat samples with a chloroform-methanol (2:1 v/v) solution and prepared in triplicate [12]. Fatty acid methyl esters were prepared prior to injection then dissolved in hexane according to the AOAC method [13]. The sample (1 μL) was injected into the gas chromatograph port by the auto sampler. The injector temperature was set at 250°C with a split ratio of 100:1. Fatty acid methyl esters were separated using a WCOT-fused silica capillary column (100 m×0.25 mm i.d., 0.20 μm film thickness; Varian Inc., Palo Alto, CA, USA) with a 1.0 mL/min helium flow. The oven was programmed as follows: 150°C/1 min, 150°C to 200°C at 7°C/min, 200°C/5 min, 200°C to 250°C at 5°C/min, and 250°C/10 min. The temperature of the detector was set at 275°C. The fatty acid peaks were identified using the retention time of fatty acid standards (47015-U, Sigma-Aldrich Corp., LLC., St. Lois, MO, USA). The peak area of each identified fatty acid was used to calculate the proportion (%) against the total identified peak area.

### Meat quality analysis

#### Instrumental surface color

The surface color was recorded by measuring the Commission International De L’eclairage (CIE) lightness (L*), redness (a*), and yellowness (b*) using a chromameter (CR-400, Konica Minolta Sensing Inc., Tokyo, Japan) with a closed cone attached. The light source of illuminant C (2° observer) with 8 mm aperture was calibrated using a white plate (Y = 93.6, X = 0.3134, y = 0.3194). Each sample was assessed at 10 different locations on the surface of the meat after blooming for 60 min in a chilling room (2°C±2°C). Chroma (C* = [a*^2^×b*^2^]^0.5^) and hue angle (h° = arctan [b*×a*^−1^]) were calculated using a data processor (DP-400, Konica Minolta Sensing Inc., Japan).

#### Meat pH and water holding capacity

For pH measurement, a total of 5 g of sample was combined with 45 mL of distilled water then homogenized at 10,000 rpm for 60 s using a homogenizer (PH91, SMT Co., Ltd., Chiba, Japan). The pH value of the homogenized meat was recorded using a pH meter calibrated with acid (pH 4.01) and neutral (pH 7.00) technical buffer solutions with an automatic temperature compensation program (SevenEasy pH, Mettler-Toledo GmbH, Greifensee, Switzerland). Water holding capacity was defined as the proportion of remaining moisture per total original moisture content before centrifugation [14]. Briefly, 5 g of ground samples were weighed into graduated centrifuge tubes, sealed and heated for 30 min in a 75°C water bath. The tubes were cooled in flowing water for 10 min then centrifuged at 980 g for 10 min at 4°C. The supernatant was decanted and measured, and the moisture contents of both raw sample and supernatant were determined according to the AOAC method [11].

#### Cooking loss and shear force

The 2.5-cm-thick samples were placed in polyethylene zipper bags and cooked in a water bath at 80°C for 45 min. The cooked samples were then immediately removed from the bags, cooled until evaporation was complete, blotted using a kitchen towel and weighed. Cooking loss was expressed as the percentage of weight loss. The cooked samples were cut (1.0 cm thick×1.0 cm width) and subjected to shear force measurement using a TA-XT2i Plus (Stable Micro Systems, Godalming, UK). Samples were cut through the slit of the table against the grain as the blade moved down with a constant speed of 200 mm/min [15]. Each assay was repeated eight times for each sample.

### Aroma volatile analysis

Samples were sliced into 3×3×2.5 cm pieces and cooked for 4 min using a skillet on a hot plate set at 165°C (Clerin, Zhongsan Tonsun Electric Appliance Co., Ltd., Guangdong, China). During cooking, samples were flipped every minute. Samples were then cooled for 5 min on aluminum foil and ground with a food blender (Hanil Electronics Co., Ltd., Korea), and aroma volatiles and patterns were immediately measured.

Aroma volatiles from cooked samples were separated and identified by gas chromatography-mass spectrometry (GC-MS) using a modified version of the method described in Ba et al [16]. Samples (3 g) were prepared in duplicate and put into 50 mL headspace vials. The vials were heated to 60°C in a drying oven for 10 min, and carboxen/polydimethylsiloxane fiber (Supelco, Sigma-Aldrich Corp., LLC., USA) with a 75 μm diameter was injected into the vial for extraction for another 30 min. Following extraction, the fiber was injected into the GC port, which was set to 250°C, and the volatiles were desorbed for 5 min at a 1:5 split ratio. Separation was performed using a DB5 fused silica column (30 m×0.25 mm inner diameter, 0.25 μm film thickness, J&W Scientific, Folsom, CA, USA) in a gas chromatograph (7890A Agilent Technologies, USA). The GC oven was programmed to operate at an initial temperature of 40°C for 2 min, increasing to 160°C (by 5°C/min), then to 180°C (by 6°C/min, holding time of 5 min), and finally to 200°C (by 10°C/min, holding time of 5 min). The interface and quadruple temperatures were 280°C and 150°C, respectively. Helium was used as the carrier gas with a flow rate of 1 mL/min. Volatile compounds were detected using a mass spectrometer (5975C, Agilent Technologies, USA). The ion source temperature of the MS was set to 280°C with an electron impact of 70 eV. A scanning mass range of 50 to 450 m/z with a scan rate of 1 scan/s was used. Tentative identification was performed by comparing the experimental spectra to the National Institute of Standards and Technology (NIST) Mass Spectral Library. Data are presented as area unit×10^6^/g.

The aroma pattern was observed using an electronic nose (FOX3000, Alpha MOS, Toulouse, France). A total of 2 g of cooked sample was placed into a 10 mL headspace vial and was prepared in duplicate. The vials were then sealed with rubber septa (Supelco 29176-U, Sigma-Aldrich Corp., LLC., USA). The samples were heated at 60°C for 600 s with an agitation speed of 500 rpm. The 2.5 mL of gas in the headspace of the samples was extracted with an automatic sampler syringe (HS 100, Alpha MOS, France) at 65°C, flow-injected into the carrier gas (synthetic air with a purity quotient >99.99%, pressure set to 0.5 bar) flow (150 mL/min) of the electronic nose and detected using a six metal oxide sensors (MOS) array system consisting of PA2, T70/2, P40/1, P10/2, P10/1, and T30/1 sensors.

### Statistical analysis

The statistical significance of the differences between means from different breeds was determined using one-way analysis of variance. Analyses were performed using R-version 3.2.0 with the “Agricolae” library (The R Foundation for Statistical Computing, Vienna, Austria). For the aroma pattern determined by electronic nose, the maximum resistance ratio was considered as the value for one measurement. Two-dimensional principal component analysis was used for data processing (Alpha Soft package version 8.01, Alpha MOS, France).

## RESULTS AND DISCUSSION

### Meat proximate and fatty acid composition

As samples were taken from carcasses of the same quality grade 1, no significant differences were found in proximate composition of strip loin between Hanwoo and Chikso beef, as shown in Table 1. The results of the present study were similar to those of a previous study, in which the fat content in quality grade 1 Hanwoo strip loin ranged from 10% to 16% [17].

Hanwoo beef has a unique fatty acid composition with abundant oleic acid (C18:1n9). The proportion of oleic acid increases as IMF content increases [17–19]. A comparison study revealed that the proportion of oleic acid in Hanwoo beef with quality grade 1++ is significantly higher than that in American crossbreed beef with choice grade [20]. However, no significant differences were found in fatty acid composition of strip loin fat between Chikso and Hanwoo beef in this study, as shown in Table 2. Overall, the fatty acid composition agreed with previous work [18]. Monounsaturated fatty acids (MUFA) were predominant in both breeds, contributing approximately 55% of total fatty acids. MUFA were followed by saturated fatty acids (SFA) and polyunsaturated fatty acids (PUFA), which contributed approximately 42% and 2.7%, respectively. Among MUFA and SFA, oleic acid had the highest contribution of up to 49% of total fatty acids, followed by palmitic (C16:0, 27%), stearic (C18:0, 10%), palmitoleic (C16:1n7, 5.8%), and myristic acid (C14:0, 3.3%), respectively. Linoleic acid (C18:2n6) was the predominant PUFA, contributing approximately 1.9% of total fatty acids. It was followed by arachidonic (C20:4n6, 0.30%), alpha-linolenic (C18:3n3, 0.10%), gamma-linolenic (C18:3n6, 0.09%), adrenic (C22:4n6, 0.08%), eicosapentaenoic (C20:5n3, 0.06%), and docosahexaenoic acid (C22:6n3, 0.04%), respectively. These results suggest that fatty acid composition is similar in Hanwoo and Chikso meat.

### Meat physical quality traits

Differences in meat physical quality traits were not observed between Hanwoo and Chikso in this study, as shown in Table 3. The surface lightness (L*) values are in line with previous study [17]. However, the redness (a*) values of our findings are slightly higher than previously reported [17]. The longer blooming time used in this study (60 min) might have contributed to these differences. Wulf and Wise [21] reported that the L* value of beef would be stable after approximately 30 min of blooming, while a* and b* values would be stable after 78 min of blooming. In general, moisture, fat and meat pH affect water-holding capacity, cooking loss and tenderness [22]. Meat physical quality traits observed in this study (e.g., instrumental surface color, water holding capacity, cooking loss, and shear force values) were not different among breeds as no differences were found in proximate composition and meat pH.

### Aroma volatiles

The volatiles released from roasted Hanwoo and Chikso beef are shown in Table 4. The total peak area of all identified volatiles was higher (p<0.02) in Chikso beef than in Hanwoo beef. Although the fat amount of Hanwoo beef was slightly higher than that of Chikso beef, the aroma intensity did not exhibit the same trend. Chikso beef released more toluene, heptanal, octanal and nonanal than Hanwoo beef. According to total peak area, toluene, heptanal, octanal and nonanal contributed 2.46%, 3.28%, 3.63%, and 3.72% of total identified volatiles, respectively, for Chikso beef. Lower proportions were observed in Hanwoo beef, in which toluene, heptanal, octanal and nonanal contributed 1.23%, 2.50% 2.19%, and 2.60% of total identified volatiles, respectively. Overall, aldehydes were predominant, in addition to other volatile groups. Hexanal and butyraldehyde were two predominant aldehydes, contributing 23.4% and 19.6% of total identified volatiles, respectively, for Hanwoo beef and 27.5% and 22.8% of total identified volatiles, respectively, for Chikso beef. Previous studies also demonstrated aldehydes to be the major volatiles in beef and showed that volatile compounds are mostly affected by a diet that alters the meat fatty acid composition [19,23,24]. Most aldehydes are the products of thermal oxidation of unsaturated fatty acids and exhibit some aroma notes such as fatty, buttery and green [4,8,25]. Although no significant differences were found in fatty acid composition, a slight difference in fat content affects the amount of each fatty acid. Higher fat content was found in Hanwoo beef, suggesting that Hanwoo beef also contained higher unsaturated fatty acids than Chikso beef. However, the estimated proportion of fat from IMF (neutral lipid) and muscle cell membrane (phospholipid bilayer or polar lipid) was not measured in this study. Wood et al [26] mentioned that the deposition of PUFA into muscle phospholipids is higher in cattle than in pig and sheep. The amount of muscle cells, which contain phospholipids, also declines when the IMF is deposited more because muscle cells and adipocytes interplay during growth [27]. As Hanwoo beef had a slightly higher fat content than Chikso beef, the tendency of bigger or wider marbling areas might be present in Hanwoo beef. In this study, a whole slice was used for pan-roasting the steak instead of a ground patty. The marbling pattern could affect the aroma development on the surface of the steak. The polar lipids in the muscle cell membrane, which contains mainly PUFA, are more prone to thermal oxidation than the neutral lipids in IMF [28]. Therefore, Chikso beef released more lipid oxidation-related aldehydes (heptanal, octanal, and nonanal) than Hanwoo beef. Further, as aldehydes content were higher than the content of other volatile groups, the area unit for total identified volatiles was higher in Chikso beef than in Hanwoo beef.

Other identified volatiles found in this study were pyrazines (2-methyl and 2,5-dimethyl), butanoic acid and 2(5H)-furanone. Pyrazines contributed 8.2% in Hanwoo and 3.9% in Chikso, butanoic acid contributed 7.6% in Hanwoo and 5.6% in Chikso, while 2(5H)-furanone contributed 6.0% in Hanwoo and 5.2% in Chikso. Meaty, roast and nutty aromas are influenced by Maillard-derived pyrazines, and the sweaty note and odor tenacity are positively correlated with the amount of butanoic acid, whereas 2(5H)-furanone is associated with sweet or caramel notes [25,29].

**Figure 1:**
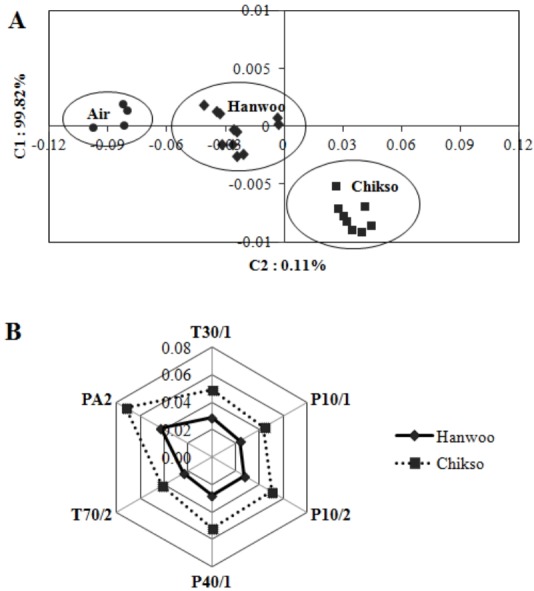
Principal component analysis of aroma pattern of roasted strip loin from Hanwoo and Chikso steers (A) and the resistance ratio of six metal oxide sensors determined using an electronic nose (B).

The aroma pattern of roasted Chikso and Hanwoo beef was well-discriminated by electronic nose (Figure 1A). The sensor resistance ratio, which was computed using a radar fingerprint chart of six MOS, is shown in Figure 1B. Figure 1A shows that Chikso beef has more distance from blank (air) than Hanwoo beef, as Chikso beef had higher resistance ratios of PA2, T70/2, P40/1, P10/2, P10/1, and T30/1 than Hanwoo beef. The total contribution rate of component 1 (C1) and component 2 (C2) was 99.82% and 0.11%, respectively. These results suggest that the aroma patterns of Hanwoo and Chikso beef differed in terms of concentration and proportion of volatiles. These findings agree with our gas chromatography results, in which Chikso beef released more intense aroma than Hanwoo beef.

## CONCLUSION

Chikso shares similar traits in terms of meat quality with Hanwoo and is as valuable as Hanwoo for beef production. However, the aroma pattern and volatiles of roasted Hanwoo and Chikso beef obtained from similar quality grade carcasses were different according to instrumental analysis results.

## Figures and Tables

**Table 1:** Proximate composition of strip loin from Hanwoo and Chikso steers finished under same diet

Variable	Hanwoo (n = 11)1)	Chikso (n = 8)1)	p value
Moisture (%)	64.8±1.51	67.4±1.36	0.23
Crude fat (%)	15.4±1.99	12.1±1.71	0.25
Crude protein (%)	18.9±0.95	19.6±0.56	0.28
Ash (%)	1.00±0.02	0.98±0.05	0.57

1) Values are presented as mean±standard error.

**Table 2:** Fatty acid composition (%) of strip loin from Hanwoo and Chikso steers finished under same diet

Compound name	Hanwoo (n = 11)1)	Chikso (n = 8)1)	p value
C14:0	3.35±0.17	3.40±0.28	0.87
C16:0	27.9±0.38	28.2±0.97	0.73
C16:1n-7	5.85±0.23	5.87±0.31	0.96
C18:0	10.4±0.33	10.3±0.32	0.78
C18:1n-9	49.8±0.58	49.5±1.39	0.83
C18:2n-6	1.96±0.12	1.94±0.11	0.88
C18:3n-6	0.09±0.01	0.10±0.01	0.26
C18:3n-3	0.10±0.01	0.11±0.01	0.37
C20:4n-6	0.31±0.04	0.32±0.04	0.86
C20:5n-3	0.06±0.00	0.07±0.01	0.25
C22:4n-6	0.08±0.01	0.08±0.01	0.65
C22:6n-3	0.04±0.00	0.05±0.01	0.37
∑SFA	41.7±0.53	41.9±1.14	0.83
∑MUFA	55.6±0.52	55.4±1.20	0.82
∑PUFA	2.65±0.16	2.68±0.13	0.89
∑n-3	0.21±0.01	0.23±0.03	0.40
∑n-6	2.44±0.16	2.45±0.14	0.98
n-6/n-3	11.6±0.77	10.6±1.50	0.89

SFA, saturated fatty acids; MUFA, monounsaturated fatty acids; PUFA, polyunsaturated fatty acids.

1) Values are presented as mean±standard error.

**Table 3:** Instrumental color and meat quality traits of strip loin from Hanwoo and Chikso steers finished under same diet

Trait	Hanwoo (n = 11)1)	Chikso (n = 8)1)	p value
CIE L*	38.4±1.21	36.9±0.69	0.34
CIE a*	23.4±0.53	24.2±0.54	0.30
CIE b*	12.8±0.30	12.9±0.34	0.91
Chroma	26.7±0.61	27.5±0.62	0.41
Hue angle	28.8±0.15	28.0±0.25	0.13
Meat pH	5.41±0.02	5.40±0.02	0.47
Water-holding capacity (%)	59.9±1.50	61.1±1.58	0.59
Cooking loss (%)	28.9±1.01	28.9±0.73	0.97
Shear force (kgf)	4.76±0.43	4.90±0.27	0.76

CIE, Commission International De L’eclairage.

1) Values are presented as mean±standard error.

**Table 4:** Aroma volatiles (area unit ×10^6^) of roasted strip loin from Hanwoo and Chikso steers finished under same diet

Compound name1)	Hanwoo (n = 11)2)	Chikso (n = 8)2)	p value
Propanal, 2-methyl	1.36±0.14	1.18±0.12	0.46
Butyraldehyde	11.9±0.74	15.9±2.58	0.89
Butanal, 3-methyl	2.55±0.37	2.46±0.37	0.22
Butanoic acid	4.64±0.40	3.90±0.16	0.81
Pentanal	2.83±0.30	2.69±0.38	0.64
Butanal, 2-methyl	8.41±1.28	7.56±0.80	0.24
Toluene	0.75±0.09	1.72±0.27	<0.01
Hexanal	14.2±3.63	19.2±2.76	0.37
Pyrazine, 2-methyl	3.15±1.02	1.43±0.18	0.29
Heptanal	1.52±0.14	2.29±0.28	0.03
2(5H)-Furanone	3.67±0.72	3.63±0.58	0.97
Pyrazine, 2,5-dimetyl	1.80±0.23	1.32±0.30	0.31
Benzaldehyde	0.95±0.07	1.29±0.18	0.14
Octanal	1.33±0.20	2.54±0.29	<0.01
Nonanal	1.58±0.10	2.60±0.45	0.04
Total	60.6±3.50	69.8±4.51	0.02

1) Compounds were identified based on mass spectra that agree with those of the Wiley Registry of Mass Spectral Data 7th ed. Agilent part No. G1035B.

2) Values are presented as mean±standard error.
